# An IoT Based Architecture for Enhancing the Effectiveness of Prototype Medical Instruments Applied to Neurodegenerative Disease Diagnosis

**DOI:** 10.3390/s19071564

**Published:** 2019-03-31

**Authors:** Alessandro Depari, Dhiego Fernandes Carvalho, Paolo Bellagente, Paolo Ferrari, Emiliano Sisinni, Alessandra Flammini, Alessandro Padovani

**Affiliations:** 1 Department Information Engineering, University of Brescia, 25123 Brescia, Italy; alessandro.depari@unibs.it (A.D.); d.fernandescarva@unibs.it (D.F.C.); paolo.bellagente@unibs.it (P.B.); paolo.ferrari@unibs.it (P.F.); alessandra.flammini@unibs.it (A.F.); 2 Neurology Unit, Department of Clinical and Experimental Sciences, University of Brescia, 25123 Brescia, Italy; alessandro.padovani@unibs.it

**Keywords:** MQTT, AMQP, medical CPS, publisher/subscriber, cloud services

## Abstract

Human errors are probably the most critical cause of the large amount of medical accidents. Medical cyber-physical systems (MCPS) have been suggested as a possible approach for detecting and limiting the impact of errors and wrong procedures. However, during the initial development phase of medical instruments, regular MCPS systems are not a viable approach, because of the high costs of repeating complex validation procedures, due to modifications of the prototype instrument. In this work, a communication architecture, inspired by recent Internet of Things (IoT) advances, is proposed for connecting prototype instruments to the cloud, to allow direct and real-time interaction between developers and instrument operators. Without loss of generality, a real-world use case is addressed, dealing with the use of transcranial magnetic stimulation (TMS) for neurodegenerative disease diagnosis. The proposed infrastructure leverages on a message-oriented middleware, complemented by historical database for further data processing. Two of the most diffused protocols for cloud data exchange (MQTT and AMQP) have been investigated. The experimental setup has been focused on the real-time performance, which are the most challenging requirements. Time-related metrics confirm the feasibility of the proposed approach, resulting in an end-to-end delay on the order of few tens of milliseconds for local networks and up to few hundreds of milliseconds for geographical scale networks.

## 1. Motivation and Introduction

The aim of a Cyber Physical System (CPS) is to integrate the computational capability of the cyber world with the real-world physical processes by means of computer networks. A CPS consists of many different intelligent entities that can dynamically interact with the environment and can cooperate and self-organize, to make autonomous decisions [1]. In the recent past, this abstract concept has been profitably moved into the industrial automation world, applying large distributed computing systems in all the steps of the manufacturing process, from the initial planning to the aftermarket, in order to improve the efficiency and lower the costs. The availability of smart objects, constituting one of the fundamental pillars of the Internet of Things (IoT) revolution, has permitted the collection of a previously inconceivable amount of data from all the sensors and actuators deployed in the field [2].

The same way smart objects of the Internet of Things (i.e., devices hosting computational and communication capabilities) surround our everyday life, smart medical devices are currently used to continuously monitor and improve the patients’ health status. From a different point of view, they can be considered a specialized version of embedded systems; they comprise sensors that sense the different parts of the patient’s body supporting diagnostic decisions by means of easy-to-use and informative operator interfaces. Very different bio-signals can be acquired and analyzed, as the electrocardiogram (ECG) [3,4], the electroencephalogram (EEG) [5], the electromyogram (EMG) [6] just to mention a few. Data provided by these instruments are subsequently complemented by physicians’ observation and constitutes a control loop with man-in-the-middle, having the medical workflow as the desired output.

As a consequence, despite several additional challenges in terms of security, dependability, privacy and safety exist when moving in the medical context, the idea of Medical CPS (MCPS) emerged as well [7,8,9,10,11,12], thanks to recent trends/needs in the medical environment.

Unfortunately, during early development stages, when only prototypes have been realized, the hardware, the internal firmware, and the procedure to use the medical instruments are not stable. Thus, MCPS medical communication busses cannot be used, due to the costs for making the instrument compliant with well-established standards. Complex certification and testing procedures must be repeated every time something inside the instrument is changed. Moreover, the MCPS communication systems have been created for satisfying the need for real-time communication on the local scale (e.g., in the intensive care units or operating rooms).

In the recent past, some of authors developed a prototype instrument for early neurodegenerative disease diagnosis [13,14]. The need for a IoT-like real-time platform for connecting several prototypes and allow remote interaction arose during the clinical trials. The complexity of the test procedure is reflected by the many parameters that can be set by the operator (usually a Medical Doctor rather than a technician). Consequently, operator expertise can greatly affect the test result, limiting the adoption of the instrument itself. When prototype instruments are sent to other research teams for test, direct interaction on a geographic scale can facilitate proper instrument usage.

The main original contributions of this work are:

- the proposal of a novel IoT-based architecture addressing requirements of prototype medical instruments, with the following characteristics: low-cost; based on well-accepted, secure, open and interoperable message oriented solutions; and able to include other information coming from ancillary sensors;
- the identification and selection of formal performance metrics for the characterization of the real-time behavior of the proposed architecture;
- the implementation of a testbed with both local and cloud servers, in order to provide close-to-reality results;
- an experimental measurement campaign for the characterization of the real-time behavior varying the location of the servers and the size of the exchanged information.

For the sake of completeness, Message Queuing Telemetry Transport (MQTT) and Advanced Message Queuing Protocol (AMQP) protocols, exploiting the publish-subscribe paradigm, have been used and compared by means of the proposed metrics. Availability, security overhead, leakage and tampering of data are out of the paper scope and have not been considered.

The paper structure is the following; in the next section, an overview of the novel technique for neurodegenerative disease diagnosis is provided, summarizing how the procedure is carried out. In Section 3, the proposed approach is detailed. In Section 4, experimental results are discussed and in Section 5 some conclusions are drawn; possible future developments are detailed as well.

## 2. Early Neurodegenerative Disease Diagnosis

The aim of this section is to provide some background information about the prototype instrument of the use case. Alzheimer’s disease (AD) and frontotemporal dementia (FTD) represent the main causes of neurodegenerative dementia in the population aged more than 60 years, with an estimated prevalence of 5% to 7%. Although it is quite easy to determine if a person is affected by dementia, early identification and classification of the specific disease is challenging (e.g., due to the fact that symptomatology is heterogeneous). Indeed, despite the AD is characterized by a well-established cholinergic impairment [15] and FTD is characterized by a GABA and glutamatergic impairment [15,16], very little has been done to develop a biomarker to identify a specific neurotransmitter.

### 2.1. Transcranial Magnetic Stimulation for Early Neurodegenerative Disease Diagnosis

However, it has been demonstrated that the impairment of different neurotransmitters, which AD and FTD selectively affect, can be effectively assessed by means of transcranial magnetic stimulation (TMS). In particular, cholinergic, GABAergic, and glutamatergic cortical circuits functionality can be indirectly evaluated by means of Short-latency Afferent Inhibition (SAI), Short-Interval Intracortical Inhibition (SICI), and Intracortical Facilitation (ICF) TMS paradigms, respectively [17,18,19]. This technique has several advantages, as this test is: (a) non-invasive, reliable, easy to apply; (b) not time-consuming, and (c) suitable as a screening tool to differentiate AD from non-AD dementia with accuracy levels comparable to currently used techniques [13,20].

TMS, originally proposed 1985, is a technique to study the motor cortex in a non-invasive and painless way; it takes advantage from an external magnetic field, penetrating the scalp and generating an induced current in the brain cortex, because of the Faraday law. Magnetic stimulus is created injecting a high current in a coil, resulting in a magnetic field up to 1–2 T, for a relatively short time (≈1 ms). In particular, the induced current allows to overcome muscle motor neuron threshold, resulting in patient’s movement that is acquired by means of electromyography (EMG). Generally, the TMS excitation probe is located in proximity of the brain cortex region, where hand muscles are considerably represented, causing a movement of patient’s fingers after a magnetic test stimulus (mTS). An adequate calibration procedure in terms of magnetic field amplitude, aiming at tuning the mTS amplitude, must be performed to obtain a patient-related reference baseline EMG response. Due to the length of the diagnosis procedure, the reference stimulus is typically repeated several times, to take into account possible baseline variations.

An mTS can be preceded by a magnetic subthreshold conditioning stimulus (mCS), sent in order to modify the patient’s response to the mTS, depending on their time distance and the action of GABAergic and glutamatergic neurotransmitters. On the contrary, if an mTS is preceded by an electrical conditioning stimulus (eCS) up to 100 V, injected by electrodes located at the patient’s wrist region, cholinergic impairment can be evaluated as well.

As a consequence, it is possible to define a stimulus train as the sequence of a conditioning stimulus followed by a test stimulus. When an mCS is followed by a mTS, a magnetic-magnetic (MM) train is defined; similarly, when an eCS is followed by a mTS, an electrical-magnetic (EM) train is defined. Finally, the reference stimulus (REF) is a particular stimulus train, where only the test stimulus (mTS) is present. The time distance between a conditioning and test stimuli in a stimulus train is referred as the inter-stimulus interval, T_ISI_. On the contrary, the time distance between the beginning of two consecutive stimulus trains is named inter-train interval, T_ITI_.

### 2.2. Diagnosis Protocol with Transcranical Magnetic Stimulation

The diagnosis protocol is a random sequence of stimulus trains, each of which characterized by a proper type (REF, MM and EM), T_ISI_ and T_ITI_. The definition of a diagnosis protocol implies the choice of the following protocol parameters:

- N_MM_: the number of distinct values of T_ISI_ that should be used in each cycle of the diagnosis phase associated with MM trains;
- a set of N_MM_ T_ISI_;
- N_EM_: the number of distinct values of T_ISI_ that should be used in each cycle of the diagnosis phase associated with EM trains;
- a set of N_EM_ T_ISI_;
- N_REF_: the number of reference stimuli, i.e., REF trains, that should be issued in each cycle of the diagnosis phase;
- T_ITI,MIN_, T_ITI,MAX_: the range of T_ITI_ to use in the diagnosis phase, to ensure aperiodic stimulation for optimal patient response;
- N_C_: the number of times that each cycle should be repeated in the diagnosis phase.

**Figure 1:**
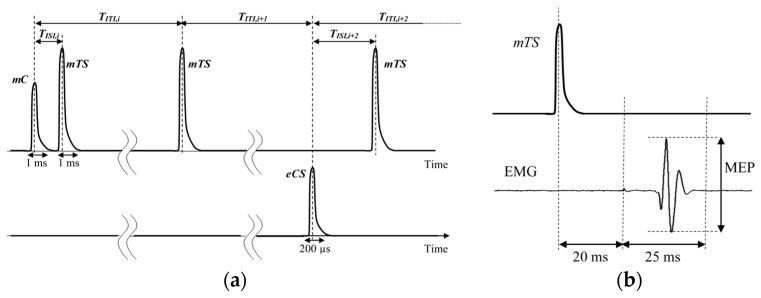
(**a**) Example of a set of three stimulus trains in a diagnosis protocol: train i is MM, train i + 1 is REF and train i + 2 is EM. The width of magnetic stimuli is usually 1 ms, whereas the width of electrical stimuli is 200 µs. T_ISI_ is usually between 1 ms and 100 ms, whereas T_ITI_ is on the order of few seconds. (**b**) Time diagram of mTS and successive EMG signal, with the quantity of interest (MEP).

Resuming, each diagnosis protocol consists on N_TOT_ = N_C_ (N_MM_ + N_EM_ + N_REF_) stimulus trains (see Figure 1a). Usually, N_MM_ ≤ 10; N_EM_ ≤ 10; N_REF_ ≤ 5 and N_TOT_ ≤ 400.

In both the diagnosis and calibration phases, the EMG should be acquired and processed to extract the quantity of interest, which is the peak-to-peak Motor Evoked Potential (MEP) measured in a time interval going from 20 ms to 45 ms after issuing the mTS, as shown in Figure 1b. The acquired MEP values are then clustered according to type and T_ISI,_ and proper statistical analysis is performed to evaluate the patient reaction to different stimulations. Then allowing specialized medical personnel to identify and to classify possible presence of dementia disease.

Due to the novelty of the test procedure, there are no ready-to-use medical instruments but rather prototype systems must be developed. Some existing commercial devices can be adopted, as reported in [13], however, the setup flexibility of these solutions is somewhat limited and very often purposely-designed prototype devices are used [14]. In all cases, the expertise of the medical personnel conducting the test is very relevant in order to ensure affordable and reliable diagnosis. For instance, repeatability can be affected by improper magnetic probe and electrode positioning, improper calibration procedure and improper diagnosis protocol definition.

### 2.3. Advantages Offered by Cloud Services

As stated in the introduction, there are well-known advantages that can be obtained digitalizing all the possible information about the test procedure. However, the supporting infrastructure is one of the key point, as described in the next section.

Regarding prototype instruments, cloud services (like storage for collecting test-related data and computing for further processing) could speed-up the innovative diagnostic procedure validation and improve its diffusion among the medical community. Particularly, a single remote supervisor (e.g., one of the researcher who developed the TMS-based instrument) could receive measurement data from many instruments all around the world (see also example shown in Figure 6). More important, since a prototype device is neither standard nor widely diffused, the effectiveness of the test procedure could be greatly improved if it was possible for the supervisor to modify the diagnosis protocol in real-time, according to his/her knowledge of the instrument behavior. Consequently, end-to-end delays compatible with typical main-in-the-loop systems are key requirements for the communication infrastructure.

In Section 3, an overview of the proposed architecture is discussed and some time-related metrics are defined. In Section 4, experimental results are detailed.

## 3. The Proposed Cloud Architecture

In order connect the TMS-based prototype instrument to the cloud, some kind of “horizontal integration” must be provided, typically in the form of middleware. In particular, smart medical devices and operators should be able to enter and leave the infrastructure as desired. The typical approach is the adoption of a message oriented middleware (MOM) based on a publisher/subscriber protocol, in which nodes announce their new data and others can selectively subscribe to them. Some standardization efforts for providing a common platform have been carried out. In the past, the IEEE11073 has been created aiming at standardizing data formats for point-to-point connections of medical instruments and systems [21,22]. Another example is the Integrated Clinical Environment (ICE) standard ASTM 2761-09, aiming at defining an interoperable environment for medical device integration, intended to improve patient safety [23]. However, despite an open source implementation exists, the OpenICE, based on the DDS—Data Distribution Service [24], its adoption is still limited, mainly due to the solution complexity. Indeed, proposals of simpler solutions exist, as the OpenICE-lite [25], leveraging the simpler Message Queuing Telemetry Transport protocol (MQTT). On the other hand, this approach suffers from the poor capability of the MQTT to customize data forwarding to subscribers and the need of an additional layer for secure transactions. These limitations are overcome using different protocols; since traceability of the test is important in medical applications and the number of nodes is relatively small, the Advanced Message Queuing Protocol (AMQP) has been identified as a viable approach; it offers higher flexibility in managing routes from the publisher to the subscriber and it is well suited for securely tracking transactions [22]. Put in other words, AMQP and MQTT are preferred to DDS due to their diffusion and easiness of implementation, that match well the needs for the prototype medical device addressed in this work. In the following, after a preliminary resume of protocol features, the proposed architecture is described and finally, some metrics for evaluating the performance are addressed.

### 3.1. The MQTT and AMQP Protocols

Both MQTT and AMQP are considered “message-centric” middleware, differently from the DDS, which is “data-centric”. Very roughly speaking, data-centric middleware offers better scalability at the cost of increasing the complexity of the middleware; furthermore, data-centric systems are well suited for complex integration problems.

The MQTT protocol is an international standard (the ISO/IEC 20922), originally proposed for exchanging machine-to-machine telemetry data in low bandwidth environments. MQTT relies on a message broker, which is a trusted intermediate in charge of ensuring message delivery from the publisher to the subscriber. Despite the acronym includes the term *Queuing*, the broker clients subscribe and publish on topics, that do not need to be previously created. Data are opaque to the broker; only the message topic is used for filtering and forwarding to the interested subscribers. The broker does not implement any store-and-forward mechanism, but offers three data delivery models corresponding to different message reliability and Quality of Service (QoS) levels. In particular, if QoS Level 0 is used, messages can be lost since there is no check of correct reception. If QoS Level 1 is chosen, a 2-way handshake mechanism is adopted, to ensure that each message is sent at least once. The QoS Level 2 implements a 4-way handshake, so that each message is sent exactly once. As a consequence, different QoS levels imply different delivery time. Regarding security concerns, MQTT relies by default on plain TCP transport protocol; however, many MQTT Brokers supports Transport Layer Security (TLS) for ciphering transactions and Simple Authentication and Security Layer (SASL) for authentication.

The AMQP-1.0 is currently an international standard (the ISO/IEC 19,464), that actively coexists with the AMQP-0.x implementations, due to the large number of available (open-source) solutions that still support the latter. In this work, without any generality loss, the AMQP-0.9.1 is considered and its main features are briefly discussed in the following. Interesting to note, the AMQP specifications include both the AMQ model, describing the components of an AMPQ-based middleware and their relationship, and the actual protocol that allow the clients to talk with the servers. In the AMQ model, the publisher sends the message to the “exchange” (a sort of mailbox) and the content of each exchange is delivered to one or more “queues” according to rules named “bindings”; the consumers subscribe to queues they are interested in. The virtual link between a publisher-subscriber pair is named “channel”. Message data are opaque to the broker (as for MQTT), except for some attributes the bindings rely on. This model makes the AMQP solution more flexible than MQTT, since the routing can be customized by the application simply changing the bindings. The QoS is related to the notion of message acknowledge, that can be automatic or manual. In the former case, the message is considered successfully delivered immediately after it is sent out (relying on the underlying transport layer for safe data delivery); in the latter, an explicit subscriber acknowledgement is required. In order to limit the traffic generated by acknowledges, a sliding-window mechanism can be implemented for manual mode, by means of a bounded channel pre-fetch threshold, that limits the number of ongoing message deliveries. Regarding the connection security, AMQP is natively based on TLS for traffic encryption and SASL for authentication.

### 3.2. The Proposed Architecture

**Figure 2:**
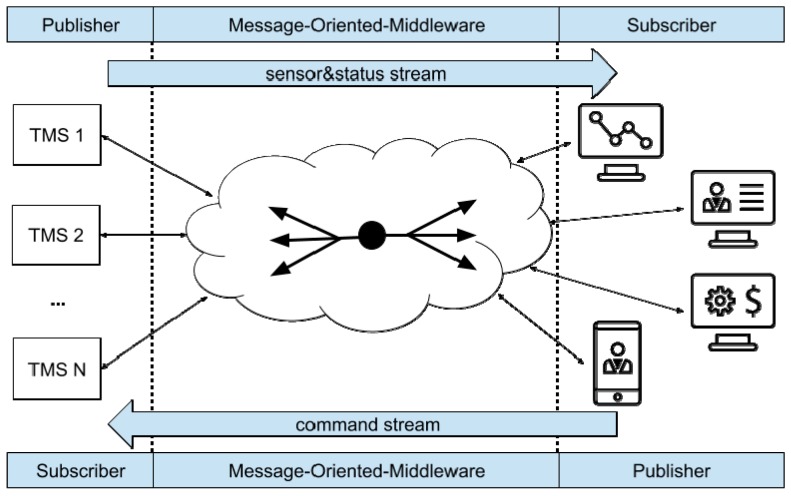
The general problem of data exchange between TMSs and their authorized users.

The general architecture of the proposed system for improving the effectiveness of prototype medical devices, as the TMS instrument previously described, is shown in Figure 2. The MOM, no matter the actual protocol, acts as a bidirectional pipe between an arbitrary number of TMS instruments on one side and an arbitrary number of users on the other one. Obviously, these end-points are not related to the actual topology of the system, but derive from the main data streams they are involved into. In particular, the *sensors measurements and systems status* data stream originates from the TMS instruments and propagates towards the end users, whereas the *commands and configurations* data stream flows in the opposite direction. The role of the MOM is to provide the proper “one-to-many” routing between the publisher (who generates the data stream) and the subscribers (which are interested in the data stream). As previously stated, publisher/subscriber paradigm offers the high flexibility and scalability required by the possibly very heterogeneous and dynamically-changing devices present in the considered scenario.

Additional services as caching (i.e., the capability to survive to network failures, as subscribers or network unavailability) and authentication and authorization (i.e., the capability to admit only trusted subscribers suitably identified) can be provided by the MOM itself or purposely-implemented as additional plugins or wrappers.

However, although the architecture shown in Figure 2 is well-suited for machine-to-machine communications, it could be cumbersome for users (e.g., medical doctors) to access data adopting plain MOM clients. Human beings are used to accessing machines via browser-based applications; for this reason, a web server directly connected with the broker is present as well. The bidirectional communication on the user side actually occurs via a websocket, thus simplifying the management of asynchronous exchanges and limiting the overall overhead and the number of TCP/IP connections per user. Websockets overcome the request/response mechanism of HTTP and permit a persistent, asynchronous connection between the client and the server. In other words, websockets start with a regular HTTP connection and then mimic a raw TCP/IP exchange in the web context.

**Figure 3:**
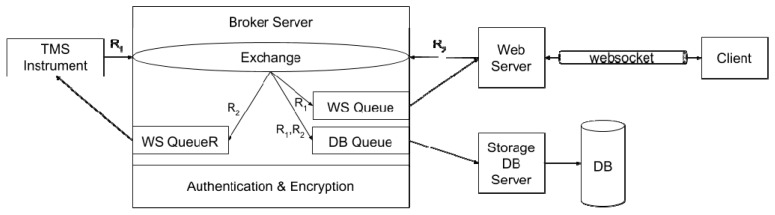
The architecture of the AMQP-based solutions. The Authentication & Encryption tier is natively supported by the AMQP protocol.

**Figure 4:**
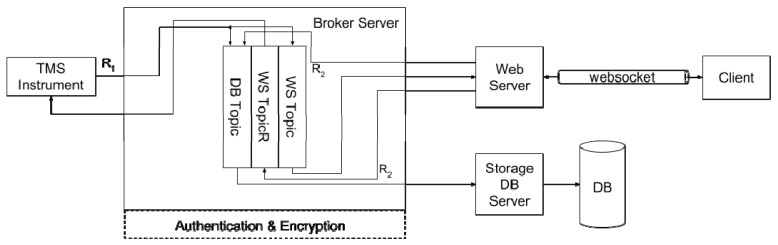
The architecture of the MQTT-based solutions. The Authentication & Encryption tier has to be implemented at higher level since it is not directly managed by the MQTT Broker.

Figure 3 and Figure 4 show the detailed architecture of the proposed framework when the AMQP and MQTT protocols are considered, respectively. For sake of generality, message storage is provided outside the broker by means of an additional database (DB), generally located in the cloud. Similarly, an authentication/security manager is in charge of handling keys and certificates. As stated before, AMQP and MQTT leverage queues and topics for relaying messages to consumers; per each TMS instrument, a set of three queues/topics is provided: the DBQueue/Topic is for storing all the message in the storage DB; the WSQueue/Topic and the WSQueueR/TopicR are for websocket exchanges. In this work, MQTT is considered as a reference implementation, due to its simplicity and wide diffusion; AMQP is considered as the actual viable solution, due to its higher flexibility and scalability.

### 3.3. The Proposed Metrics for Performance Evaluation

In order to assess and verify the performance of a communication infrastructure, a suitable set of metrics must be defined. For instance, in the past, some efforts were carried out for measuring the Internet infrastructure at the network level [26]; metrics typically considered include end-to-end or OWD (one-way delay) and delay variations [27,28]. Referring to the Internet characterization, the RFC2679 defines the OWD as the time elapsing from the occurrence of the first bit of a packet at the source and the occurrence of the last bit of a packet at the destination; the RFC3393 introduces the Inter Packed Delay Variation (IPDV, i.e., the jitter), computed as the difference between the OWD of a selected pair of packets in a test stream. However, since the actual timing requirements and constrains depend on the application, the delay evaluation (and possible control) should be performed at the application level [29,30,31,32,33,34,35].

**Figure 5:**
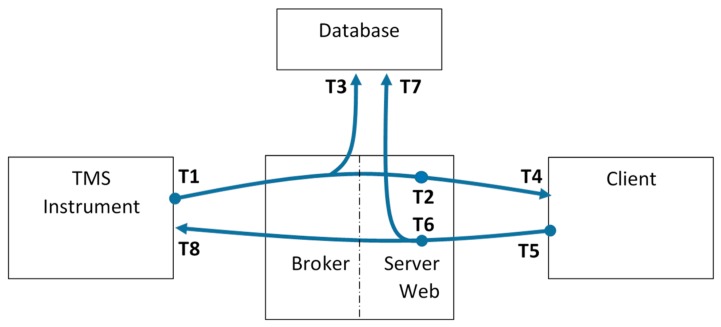
The different timestamps collected for evaluating the proposed time-related metrics.

This paper is mainly focused on the real-time availability of data regarding the medical device; in this way, the information coming from the prototype instruments will be transparently shared among local and remote users. For this reason, a set of metrics related to the end-to-end delays introduced by the proposed architecture are defined. The delays can be calculated only if precise timestamps are taken at relevant events, like data/command transfers. Hence, the following timestamps are defined (see Figure 5):

- T1: this timestamp is assigned by the instruments when a new set of data is ready to be sent to the architecture.
- T2: this timestamp is assigned by the architecture when a new set of data arrives to the Web Server coming from the instrument.
- T3: this timestamp is assigned by the database when a new set of data coming from the instruments is permanently stored in its data table.
- T4: this timestamp is assigned by the Client when a new set of data arrives through the websocket.
- T5: this timestamp is assigned by the Client when a new command leaves the Client addressed to the architecture.
- T6: this timestamp is assigned by the architecture when a new set of data arrives to the Web Server coming from the Client.
- T7: this timestamp is assigned by the database when a new set of data coming from the Client is permanently stored in its data table.
- T8: this timestamp is assigned by the instrument when a command arrives through the architecture.

Using these timestamps, the proposed consistent set of metrics is:

- **DIC** = T4–T1: the overall end-to-end delay from the TMS instrument to the remote Client.
- **DIS** = T2–T1: the end-to-end delay from the TMS instrument to the Web Server.
- **DID** = T3–T1: the end-to-end delay from the TMS instruments to the Database.
- **DCI** = T8–T5: the reverse overall end-to-end delay from the remote Client to the TMS instrument.
- **DCS** = T6–T5: the end-to end delay from the Client to the Web Server.
- **DCD** = T7–T5: the end-to-end delay from the Client to the Database.

## 4. Validation and Experimental Results

The feasibility of the proposed architecture is investigated in this section. The different actors identified in the previous section are “black boxes” from the user’s point view; accordingly, the overall metrics of interest can be evaluated creating simulated scenarios [36,37] or using specific experiments, as in [38,39]. The latter approach has been pursued in this work. The used instruments and the experimental setup are introduced and characterized in specific subsections. Last, the experimental results are presented and discussed.

### 4.1. The Prototype Medical Instrument for Neurodegenerative Disease Diagnosis

As stated before, the aforementioned TMS-based technique for neurodegenerative disease diagnosis is currently applied by means of purposely-designed and ad-hoc implemented excitation/acquisition systems. According to the operating principle, the minimal requirements include a magnetic and electric stimuli generator and an EMG acquisition system.

The magnetic excitation probe, suitable for cortical stimulation and monophasic waveforms, typically has a figure-of-eight shape and hosts a pair of coils, each of which with a diameter on the order of 100 mm. The operator handhelds the probe, so that the center is in contact with the patient’s scalp and the coils are tangent with the scalp surface. The operator is in charge of ensuring the alignment with the motor cortex gyrus, i.e., the correct positioning of the coil involves movements in the antero-posterior and latero-lateral plains as well as rotation around the axis perpendicular to the scalp surface (typically 45° from the midline). As previously described, magnetic induction field is up to 2 T at the probe surface.

Bar electrodes are used for injecting electrical stimuli. They are typically applied at the patient’s arm, few centimeters apart from each other. In particular, the bar electrodes should be placed at the wrist’s internal side, over the median nerve, longitudinally oriented with the cathode positioned proximally.

Finally, MEP signals, obtained via surface EMG, are collected by means of single pair of Ag/AgCl electrodes, that should be placed on the first interosseous muscle, dorsal side, and metacarpal-phalangeal joint, lateral side, respectively. A third electrode should be used to provide a proper reference voltage to the user’s wrist, dorsal side.

Magnetic and electrical actuating elements are connected to stimulators (drivers), whereas EMG sensing elements are connected to an amplifier. The magnetic stimulator should be able to issue REF stimulus trains (i.e., a single mTS) as well MM stimulus trains (i.e., an mCS followed by an mTS). Moreover, in case of EM stimulus trains (i.e., an eCS followed by an mTS), the magnetic stimulator takes care of generating the mTS, suitably synchronized with the eCS. The amplitude of the mTS and mCS stimuli should be independently tunable in step of 1% with respect to the maximum value. The electric stimulator should be able to issue the single electrical unipolar pulse eCS for the EM stimulus trains. The electrical stimulus should have a constant width of 200 µs (with a resolution of 10 µs) and magnitude should be tunable between 0 and 100 V in steps of 1 V. T_ISI_ interval, for both MM and EM stimulus trains, should be tunable between 1 ms and 250 ms in steps of 1 ms (with a resolution of 100 µs). The inter-train interval T_ITI_ should be tunable in steps of 100 ms, with a typical range from 4 to 10 s. The EMG amplifier should have an input range of at least 10 mVpp and a sampling frequency of at least 1 kHz (the minimum MEP bandwidth is typically 10 Hz–500 Hz); vertical resolution should be 10 µV (i.e., Effective Number of Bits—ENOB—10 bit). The EMG amplifier should be able to provide a proper ground voltage by means of the reference electrode. Commercial drivers and amplifiers are available; in this work a BISTIM^2^ system (from Magstim, Whitland, UK) for magnetic stimulation and a STMISOLA (from Biopac, Goleta, CA, USA) for electrical stimulation are used; a QUATTRO device (from Otbioelettronica, Torino, Italy) has been employed as electromyograph.

A controller unit is needed to manage all the blocks of the system. The controller is in charge of: correctly configuring and synchronizing the stimulators, to provide the proper sequence of stimuli (the diagnosis protocol); processing raw EMG data; and managing the web-based user interface for the local operator. In particular, the stand-alone operation of the instrument requires a minimum set of functionalities, including: the selection of operation mode (among diagnosis, magnetic calibration, electric calibration); issuing of start and stop commands for diagnosis and calibration phases; real time visualization of MEP value for the calibration phases; tuning of magnetic and electrical stimuli amplitude; visualization of the automatic response of data analysis after the diagnosis phase; and stimuli randomization.

Some of the authors developed a prototype controller, the core of which is based on a single-board computer running Linux (a Raspberry Pi board, chosen due to the compactness, computational capability, peripheral availability, and wide support from the user community). On the contrary, the synchronized management of the stimuli trigger signals (according to the diagnosis protocol previously described) has been assigned to a more deterministic Field Programmable Gate Array unit (FPGA, in particular a Cyclone III EP3C16F from Altera, San Jose, CA, USA).

In this work, the controller has been enhanced to further support the IoT-like framework detailed in Section 3. In particular, the system is able to provide raw data about the diagnosis protocol via AMQP (and MQTT) protocol. According to the previous statements, the useful information includes:

- protocol invariant data, as the amplitude of the stimuli, A_mTS_, A_mCS_, A_eCS_; each of which is one byte wide;
- an array of N_TOT_ records, each of which specifying the stimulus type (1 byte), the T_ISI_ value (1 byte), the T_ITI_ value (1 byte), and the calculated MEP value (2 byte).

Since N_TOT_ ≤ 400, the acquisition of a whole diagnosis protocol requires less than 2 KB. Additionally, the system is able to provide raw samples of the EMG signal, i.e., 25 samples 2 byte wide per each cycle; the overall EMG data require about 5 KB.

**Figure 6:**
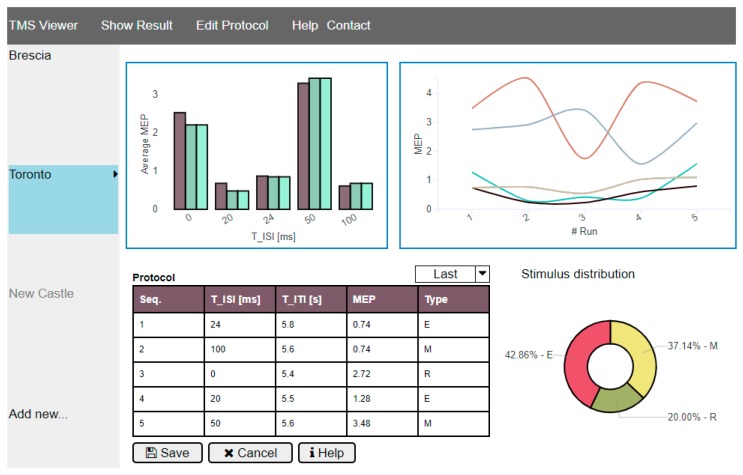
Client dashboard for remote supervision of diagnosis protocol carried out with the prototype TMS instruments.

For sake of completeness, in Figure 6 a possible dashboard illustrating data available after a diagnosis protocol has been completed is shown. As stated in the introduction, the proposed solution allows a remote supervisor to interact in real-time with TMS-based instrument located all around the world. In particular, the current measurement results are presented in real-time in the “Show Result” page, whereas the supervisor can change the diagnosis protocol to be applied in the “Edit Protocol” page.

### 4.2. Experimental Setup

**Figure 7:**
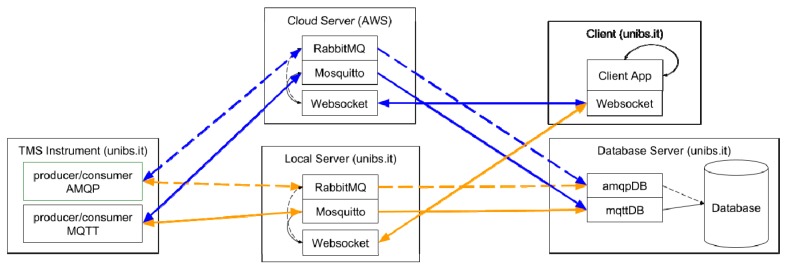
Block diagram of the implemented experimental setup.

The experimental setup is depicted in Figure 7. There are five main actors involved in the experiments. The designed experiment requires a message starting from the TMS Instrument to travel toward the Client, being saved also in the database; in the Client, a loopback mechanism triggers the sending of a command message that travels back to the TMS Instrument, activating the saving action inside the database as well. AMQP and MQTT Brokers are implemented using open source solutions, RabbitMQ (version 3.7.8) and Mosquitto (version 1.5.3), respectively. They have been chosen due to their diffusion and availability for many different platforms. RabbitMQ is written in Erlang and has been developed within the Open Telecom Platform framework for failover and clustering. RabbitMQ natively supports AMQP-0.9.1 as the core protocol and offers a gateway for HTTP enabling a management plugin, mainly for diagnostic purposes, other than supporting messaging services toward a browser based on STOMP over websocket and JSON-RPC. Mosquitto is an Eclipse project; it implements MQTT version 3.1.1 and it is written in C. It has been mainly designed for satisfying lightweight messaging applications, especially when computational capabilities are scarce.

Each actor in the experiment has its own characteristics, as listed in the following:

- The TMS Instrument is based on a Raspberry Pi 2 single board computer, as described in Section 4.1. The raspberry Pi 2 hosts a quad-core 64 bit ARM processor (clock frequency 1.2 GHz), 1 GB of RAM, four USB ports, a LAN interface and HDMI video connection; it runs the Linux-based Raspbian OS. The TMS Instrument role is to initiate the experiment, sending the first data message. It also terminate the experiment, receiving the Client command. A custom transport layer abstracting the communication libraries for both AMQP and MQTT has been implemented in Python; in particular, the transport layer uses the Paho library for MQTT and the Pika library for AMQP.
- The Client represents the remote operator that needs to interact with the prototype TMS Instrument. In this experiment, the simple Client role is to receive data from the instrument and loopback them into a command for the instrument. It has been implemented with an embedded device to introduce the minimum overhead. The device is a Siemens IOT2040 (based on an Intel Quark × 1020 (+Secure Boot) processor and hosting 1 GB of RAM, two Ethernet ports and two RS232/485 interfaces, and a battery-backed Real Time Calendar. It runs a customized Yocto Linux distribution. The entire software at client side is written in Javascript and runs in Node.Js, a well-accepted approach in IoT solutions that ensures portability on different platforms. The Client is located inside the domain of the University of Brescia.
- The Local Server is a machine specifically created for the experiments. It is located inside the domain of the University of Brescia. It is a VMware (ESXi 6.5) virtual machine (VM); the VM is hosted on a DELL PowerEdge R630 (Intel Xeon E5–2 core, 128 GB RAM, 4 × 1 Gbps Ethernet), one of the facility available in the University of Brescia eLUX laboratory [40]. The VM has a single CPU, 2 GB of RAM and has CentOS7 as guest operating System (OS). It hosts the RabbitMQ AMQP broker, with the HTTP management plugin enabled, and the Mosquitto MQTT broker. The local server hosts also the websocket server, which is a simple Python 3.6 program. It uses the Tornado web server websocket implementation to communicate with the Client and it runs the same custom transport layer used in the TMS Instrument (which, as previously stated, leverages the Paho library for MQTT and the Pika library for AMQP).
- The Cloud Server is an instance of the Amazon AWS EC2 t2.micro virtual machine with a single CPU and 1 GB of RAM. The Cloud Server is hosted by the US East (Ohio) Amazon Web Service (AWS) data center and it has been specifically created for the experiments. The software configuration of the Cloud Server is identical to the Local Server one; a minimal effort is required for migration from Local to cloud Server.
- The Database Server is located inside the domain of the University of Brescia. It is a VMware (ESXi 6.5) VM; it is hosted on a Syneto Ultra 205 hyperconverged system (Intel Xeon E5–6-core, 64 GB RAM, 4 × 1 Gbps Ethernet), another facility available in the eLUX laboratory. The VM has 4 CPU, 16 GB RAM and has CentOS7 as guest OS. It hosts the different databases (including MariaDB, Influxdb, PosgreSQL) that collect the laboratory data. For the experiments of this work, the Influxdb, an open-source database purposely-designed for time series storage, has been chosen.

The experimental setup is easily configurable to change the message protocol (AMQP or MQTT) and the location of the Server. As shown in Figure 7, the AMQP scenario is based on the AMQP blocks inside the actors, with the dashed lines showing the message exchange paths. On the other hand, the solid lines connect the MQTT blocks for the implementation of the MQTT scenario. The local and cloud scenario are denoted by different line color: orange for Local Servers, blue for Cloud Server. In all the experiments, default configuration of Brokers is considered, if not explicitly stated otherwise; due to the previously highlighted constraints, message size is set to 2 KB, representative of typical information transferred per diagnosis protocol, as stated in Section 4.2.

### 4.3. Estimation of Measurement Uncertainty

**Figure 8:**
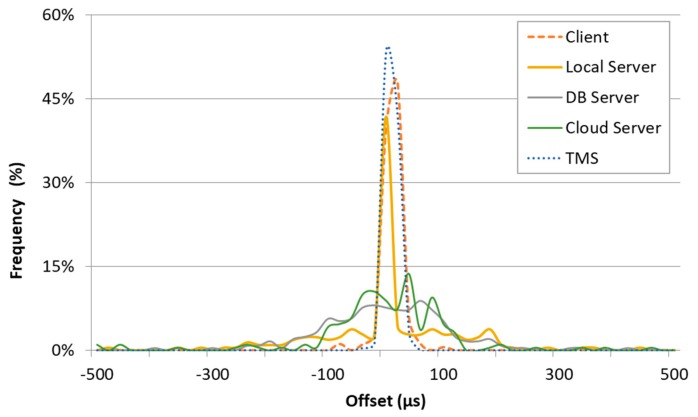
Probability Density Function (PDF) estimate of the residual offset after NTP daemon corrected the local system clock of the devices involved in the experiments.

To calculate the metrics defined in the previous section, a synchronization mechanism between all the devices/machines involved in the distributed system is necessary. In this paper, the NTP synchronization is used. It is periodically executed in every device and the local system clock is corrected using the offset and delay measurements from a global NTP server, which is, in turn, synchronized to the UTC source. The uncertainty of the time synchronization using NTP varies with the quality of the Internet connection in terms of latency, and it is typically on milliseconds scale [41] The NTP daemon transparently calculates and applies corrections, logging error statistics when requested. In this paper, the synchronization uncertainty has been evaluated using the residual offset of the system clock at step N, which is the residual error after the clock corrections calculated at step N-1 have modified the local clock behavior. The results are shown in Table 1 and Figure 8 for a one-day observation time, compatible with the observation time used to evaluate the experiments.

There is a systematic error introduced by the NTP software which is hard to be compensated by calibration procedures. In this paper, the experimental synchronization standard uncertainty $u_{sn}$ takes into account all the error components: the average error $\mu_{sn}$ and the standard deviation $\sigma_{sn}$. For this reason, the experimental synchronization standard uncertainty has been evaluated as $u_{sn}=\sqrt{\mu_{sn}^{2}+\sigma_{sn}^{2}}$ [42].

From Table 1, the synchronization uncertainty (with respect to the UTC) of all the devices involved in the experimental setup is less than 0.3 ms. In the proposed experimental setup, the event timestamping is carried out in software. This situation introduced an uncertainty contribution which is evaluated with a specific experiment, the full description of which is given in [34] and briefly resumed here. The timestamp uncertainty is estimated executing a software loop that collects a timestamp just before (T9) and just after (T10) a routine that creates a fixed and known delay of value K. In theory, the timestamps are taken from the same system clock used to calculate the delay, so the quantity Δ=T10−T9−K should be equal to zero. In practice, the experimental results show a variability and the uncertainty $u_{\Delta }$ of the quantity Δ can be modelled as $u_{\Delta }=\sqrt{\mu_{\Delta }^{2}+\sigma_{\Delta }^{2}}$, because both standard deviation $\sigma_{\Delta }$ and the average value $\mu_{\Delta }\;$(i.e., the systematic error) are considered. Supposing that the uncertainty $u_{\Delta }$ is completely due to the timestamp uncertainty $u_{tn}$ that disturbs both T8 and T9, $u_{tn}$ can be estimated with the Equation (1):

(1) $$u_{\Delta }^{2}=u_{tn}^{2}+u_{tn}^{2}=2u_{tn}^{2}\to u_{tn}=\sqrt{\frac{u_{\Delta }^{2}}{2}},$$

The results (over 1000 samples) are shown in Table 2 for all the devices involved in the experiments; the maximum timestamp standard uncertainty *u_tn_* is on the order of 3 ms for the Client machine, whereas all the other devices show uncertainty under 1 ms.

It has to be noted that: all the devices are able to synchronize with a very low average offset (Table 1), and the timestamp uncertainty is dominating over the synchronization uncertainty.

The metrics described in Section 3.3 involve the timestamps taken in different devices. The uncertainty $u_{M}$ of each metric is different, depending on the uncertainty of the devices involved in its calculation. Equation (2) can be used to estimate the uncertainty of the measurements. The resulting uncertainty values are reported in Table 3 for all the metrics to be calculated, when the synchronization uncertainty and the timestamp uncertainty of Table 1 and Table 2 are considered. In details, being A and B two devices used for the metric calculation, the relation expressing the combination of the uncertainties is the following:

(2) $$u_{M}=\;\sqrt{u_{sA}^{2}+u_{tA}^{2}+u_{sB}^{2}+u_{tB}^{2}},$$

As expected, due to the squaring operation, the main contribution dominates and, analyzing Table 3, it is clear that the metric uncertainty is greater when a timestamp taken by the Client is involved in the metric calculation.

### 4.4. Experimental Results

Each single experiment is aimed at collecting all the relevant timestamps along the path described in Section 4.2. More than 200 samples are collected for each experiment run. The experiment campaign is composed of eight scenarios, obtained varying the following parameters:

- interval between data transfer (1 s or 10 s);
- location of Broker and Web Server (Local Server or Cloud Server);
- the used message protocol (AMQP or MQTT).

The transferred data is 2 KB per message and the DB server and the Client are in the same local network. The metrics defined in Section 3.3 have been calculated for each scenario and are shown in Table 4.

First of all, it can be affirmed that the AMQP and the MQTT implementations practically introduce similar delays, even if the number of features offered by the two brokers is different. Additionally, from the previous results, it is clear that, when the cloud scenario is considered (messages passing twice across geographical networks, i.e., from the TMS instrument to the cloud server and then from the cloud server to the database server or the client), an additional delay of about 130 ms is added. Nevertheless, the 95 percentile of the end-to-end delay is always less than 200 ms, so that the proposed communication framework can satisfy real-time requirements of supervision/control applications with human in-the-loop. Finally, there is an asymmetry between the data flow (from the TMS instrument to the operator) and the commands flow (the opposite direction); it has to be attributed to the different behavior of the experimental websocket server managing input or output streams, since websocket sessions are mostly symmetrical.

**Figure 9:**
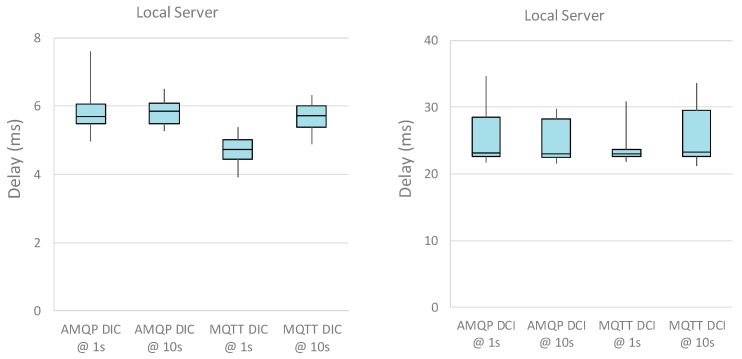
Boxplot of the DIC and DCI performance metrics with local server. The plot shows a blue box from the 25 percentile to the 75 percentile (with the median line). The whiskers go from the 5 percentile up the 95 percentile.

**Figure 10:**
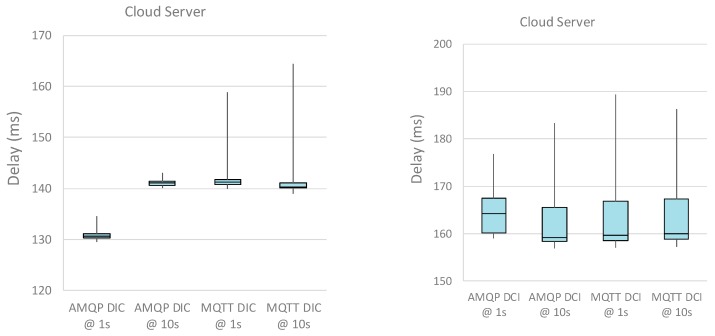
Boxplot of the DIC and DCI performance metrics with cloud server. The plot shows a blue box from the 25 percentile to the 75 percentile (with the median line). The whiskers go from the 5 percentile up the 95 percentile.

For sake of completeness, the boxplots of the DIC and DCI performance metrics for different intervals between data transfer are shown in Figure 9 and Figure 10, for local and cloud server implementations, respectively. The boxplots highlight the distribution of the samples and, in this specific case, it is evident the larger support of the distributions related to the cloud server (as expected). Last, it can be argued that standard deviation of the metrics is (often) greater than the measurement uncertainty, highlighting the additional variability introduced by the whole system under test.

**Figure 11:**
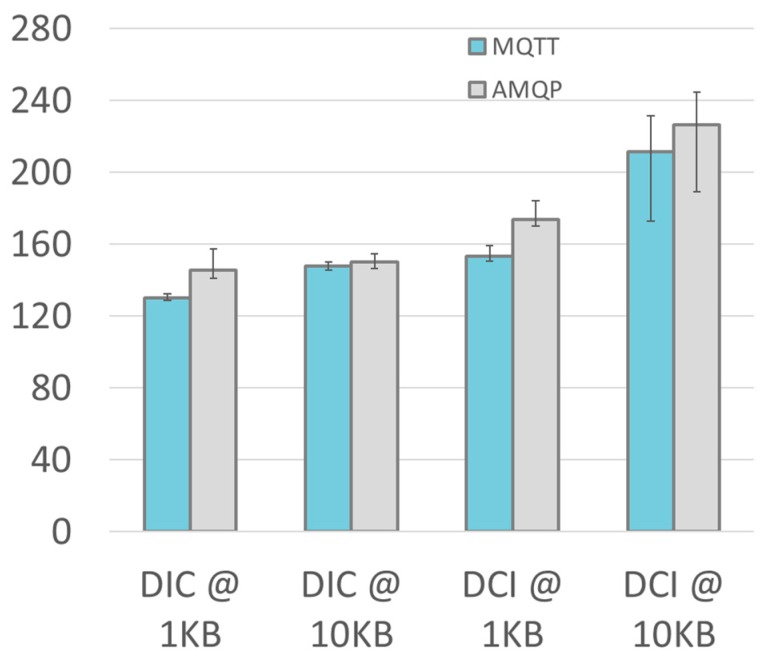
Bar graph of the DIC and DCI performance metrics with cloud server. The whiskers go from the 5 percentile up the 95 percentile.

Another experiment has been designed to understand the relation between the size of the exchanged information and the end to end delay. In this experiment, the DIC and DCI metrics have been considered in the case of cloud server (which is the most demanding). The results for size varying from 1 KB to 10 KB are graphically presented in Figure 11. As expected, the size of data has a direct impact on the DIC and DCI. The delay grow is higher for MQTT when the size increases, whereas the variability is almost the same between AMQP and MQTT.

In conclusion, considering all the experimental evidences, the AMQP solutions offers many more features (e.g., security and authentications) with respect to the MQTT basic implementation, without sacrificing the performance. For this reasons, AMQP seems to be the best solution for the implementation of the proposed architecture.

## 5. Conclusions and Future Development

Cyber-physical systems are characterized by the close and tight coupling between the real-world physical components (with their own dynamics) and the digital counterpart aimed at monitoring and controlling. The interaction between the two domains requires completely new communication paradigms, which are changing the way humans interact with and control the surrounding environment. CPSs must satisfy stringent requirements in terms of dependability, security, safety, and efficiency in real time, while satisfying privacy constraints. Healthcare and medical devices and systems have been transformed into MCPSs, taking advantage of improved data sharing capabilities. In this work, inspired by recent IoT advances, a simple real-time solution to address peculiarities of prototype medical diagnostic tests, which are often based on proof-of-concept instruments requiring a high expertise level by the operators, is proposed. The focus is an innovative technique for early diagnosis of dementia based on Transcranial Magnetic Stimulation, but the proposed approach may be applied for different diagnostic solutions at the prototype stage. Authors demonstrated that the use of a message oriented middleware allows to easy interconnect in real-time several prototype instruments and medical personnel on both local and geographical scale. In particular, when widely diffused and well-accepted protocols as AMQP and MQTT are adopted, it has been experimentally verified that the end-to-end delay of the communication framework is compatible with real-time requirements of medical control applications with a human-in-the-loop. Moreover, the preference goes to the AMQP solutions: the considered scenario, AMQP has the same performance of MQTT and it natively offers more features (like authentication). A purposely-designed experimental setup has been implemented to evaluate time-related metrics on both a local and geographical scale. Thanks to the proper design of the infrastructure, moving from local to geographical scale is almost straightforward. The synchronization capability has been verified, resulting in a worst-case synchronization uncertainty on the order of 3 ms. The overall end-to-end delay from the TMS instrument to the remote client has an average value of about 140 ms, which rises up to about 160 ms for the opposite direction, confirming the feasibility of the proposed approach. As a concluding remark, the experiments highlight that, when the geographical scale is considered, the cloud service provider may greatly impact on performance, no matter the peculiar implementation.

In the future, the proposed solution could be profitably used for exchanging not only raw test samples (to be further processed in the cloud), but ancillary meta-data as well (e.g., images or small videos of the testing procedure, real-time location of the personnel and patient involved in the test, serial/identification numbers of the adopted instrumentation, etc.). For these reasons, specific plugins implementing typical MCPS interface (e.g., OpenICE) are under investigation.

## Figures and Tables

**Table 1:** Synchronization uncertainty of the devices involved in the experiments (ms).

Device	*u_sn_*	$\mu_{\mathrm{sn}}$	$\sigma_{\mathrm{sn}}$
TMS Instrument	0.009	0.000	0.009
Local Server	0.172	0.007	0.172
Cloud Server	0.278	0.013	0.278
DB Server	0.126	0.011	0.125
Client	0.018	0.001	0.018

**Table 2:** Timestamp uncertainty of the devices involved in the experiments (ms).

Device	*u_tn_*	$\mu_{\Delta }$	$\sigma_{\Delta }$
TMS Instrument	1.0	1.4	0.2
Local Server	0.7	1.0	0.1
Cloud Server	0.8	1.1	0.3
DB Server	0.8	1.0	0.3
Client	3.1	4.3	0.9

**Table 3:** Synchronization uncertainty related to the metrics used in the experiments (ms).

	Metric	Device A	Device B	$u_{M}$
Local	TMS to Client	TMS Instrument	Client	3.3
	TMS to Server	TMS Instrument	Local Server	1.2
	TMS to Database	TMS Instrument	DB Server	1.3
	Client to TMS	Client	TMS Instrument	3.3
	Client to Server	Client	Local Server	3.2
	Client to Database	Client	DB Server	3.2
Cloud	TMS to Client	TMS Instrument	Client	3.3
	TMS to Server	TMS Instrument	AWS Server	1.3
	TMS to Database	TMS Instrument	DB Server	1.3
	Client to TMS	Client	TMS Instrument	3.3
	Client to Server	Client	Cloud Server	3.2
	Client to Database	Client	DB Server	3.2

**Table 4:** Performance metrics of the proposed architecture as implemented for the experiments (ms).

	Metric	MQTT @ 1 s		AMQP @ 1 s		MQTT @ 10 s		AMQP @ 10 s	
		Mean	95 perc.	Mean	95 perc.	Mean	95 perc.	Mean	95 perc.
Local	D_IC_	4.8	5.4	7.0	7.6	6.3	6.3	6.2	6.5
	D_IS_	0.5	0.9	2.0	2.3	1.7	1.8	2.0	2.3
	D_ID_	2.3	3.5	3.8	4.3	3.4	4.1	3.8	4.4
	D_CI_	25.7	30.9	27.8	34.6	26.2	33.5	25.0	29.8
	D_CS_	22.1	25.4	24.1	30.6	22.7	26.4	21.2	25.2
	D_CD_	25.4	30.7	28.6	35.5	28.2	37.3	25.9	30.8
Cloud	D_IC_	144.6	158.9	132.5	134.6	145.0	164.3	141.8	143.0
	D_IS_	71.4	71.9	59.8	60.3	71.9	72.9	72.0	72.7
	D_ID_	141.9	147.7	128.1	129.0	141.1	148.9	140.3	141.9
	D_CI_	164.3	189.4	168.5	176.7	165.9	186.3	164.0	183.3
	D_CS_	93.7	111.8	97.5	103.8	94.1	110.6	94.5	115.3
	D_CD_	164.3	189.5	169.3	178.2	165.8	186.8	166.5	185.5

## References

[1] Khaitan S.K., McCalley J.D. (2015). Design Techniques and Applications of Cyberphysical Systems: A Survey. IEEE Syst. J..

[2] Al-Fuqaha A., Guizani M., Mohammadi M., Aledhari M., Ayyash M. (2015). Internet of Things: A Survey on Enabling Technologies, Protocols, and Applications. IEEE Commun. Surv. Tutor..

[3] Crema C., Depari A., Flammini A., Sisinni E., Vezzoli A., Bellagente P. (2017). Virtual Respiratory Rate Sensors: An Example of a Smartphone-Based Integrated and Multiparametric mHealth Gateway. IEEE Trans. Instrum. Meas..

[4] Depari A., Flammini A., Sisinni E., Vezzoli A. A wearable smartphone-based system for electrocardiogram acquisition. Proceedings of the Medical Measurements and Applications (MeMeA) 2014 IEEE International Symposium.

[5] Zhang X., Li J., Liu Y., Zhang Z., Wang Z., Luo D., Zhou X., Zhu M., Salman W., Hu G. (2017). Design of a Fatigue Detection System for High-Speed Trains Based on Driver Vigilance Using a Wireless Wearable EEG. Sensors.

[6] Zia ur Rehman M., Waris A., Gilani S.O., Jochumsen M., Niazi I.K., Jamil M., Farina D., Kamavuako E.N. (2018). Multiday EMG-Based Classification of Hand Motions with Deep Learning Techniques. Sensors.

[7] Ivanov R., Weimer J., Lee I. Context-Aware Detection in Medical Cyber-Physical Systems. Proceedings of the International Conference on Cyber-Physical Systems (ICCPS).

[8] Jezewski J., Pawlak A., Wróbel J., Horoba K., Penkala P. (2015). Towards a medical cyber-physical system for home telecare of high-risk pregnancy. IFAC-PapersOnLine.

[9] Lee I., Sokolsky O., Chen S., Hatcliff J., Jee E., Kim B., King A.L., Mullen-Fortino M., Park S., Roederer A. (2012). Challenges and Research Directions in Medical Cyber–Physical Systems. Proc. IEEE.

[10] Kocabas O., Soyata T., Aktas M.K. (2016). Emerging Security Mechanisms for Medical Cyber Physical Systems. IEEE/ACM Trans. Comput. Biol. Bioinform..

[11] Dey N., Ashour A.S., Shi F., Fong S.J., Tavares J., Manuel R.S. (2018). Medical cyber-physical systems: A survey. J. Med. Syst..

[12] Li T., Cao J., Liang J., Zheng J. (2015). Towards context-aware medical cyber-physical systems: Design methodology and a case study. Cyber Phys. Syst..

[13] Padovani A., Benussi A., Cantoni V., Dell’Era V., Cotelli M.S., Caratozzolo S., Turrone R., Rozzini L., Alberici A., Altomare D. (2018). Diagnosis of Mild Cognitive Impairment Due to Alzheimer’s Disease with Transcranial Magnetic Stimulation. J. Alzheimer’s Dis..

[14] Crema C., Depari A., Sisinni E., Benussi A., Borroni B., Padovani A. Embedded platform-based system for early detection of Alzheimer disease through transcranial magnetic stimulation. Proceedings of the 2018 IEEE Sensors Applications Symposium (SAS).

[15] Francis P.T., Palmer A.M., Snape M., Wilcock G.K. (1999). The cholinergic hypothesis of Alzheimer’s disease: A review of progress. J. Neurol. Neurosurg. Psychiatry.

[16] Levenga J., Krishnamurthy P., Rajamohamedsait H., Wong H., Franke T.F., Cain P., Sigurdsson E.M., Hoeffer C.A. (2013). Tau pathology induces loss of GABAergic interneurons leading to altered synaptic plasticity and behavioral impairments. Acta Neuropathol. Commun..

[17] Tokimura H., Di Lazzaro V., Tokimura Y., Oliviero A., Profice P., Insola A., Mazzone P., Tonali P., Rothwell J.C. (2000). Short latency inhibition of human hand motor cortex by somatosensory input from the hand. J. Physiol..

[18] Kujirai T., Caramia M.D., Rothwell J.C., Day B.L., Thompson P.D., Ferbert A., Wroe S., Asselman P., Marsden C.D. (1993). Corticocortical inhibition in human motor cortex. J. Physiol..

[19] Ziemann U., Rothwell J.C., Ridding M.C. (1996). Interaction between intracortical inhibition and facilitation in human motor cortex. J. Physiol..

[20] Benussi A., Di Lorenzo F., Dell’Era V., Cosseddu M., Alberici A., Caratozzolo S., Cotelli M.S., Micheli A., Rozzini L., Depari A. (2017). Transcranial magnetic stimulation distinguishes Alzheimer’s disease from Frontotemporal Dementia. Neurology.

[21] Blumrosen G., Avisdris N., Kupfer R., Rubinsky B. C-SMART: Efficient seamless cellular phone based patient monitoring system. Proceedings of the 2011 IEEE International Symposium on a World of Wireless, Mobile and Multimedia Networks.

[22] Bellagente P., Depari A., Ferrari P., Flammini A., Rinaldi S., Sisinni E. M3IoT-Message-oriented middleware for M-health Internet of Things: Design and validation. Proceedings of the 2018 IEEE International Instrumentation and Measurement Technology Conference (I2MTC).

[23] Larson B.R., Zhang Y., Barrett S.C., Hatcliff J., Jones P.L. (2015). Enabling Safe Interoperation by Medical Device Virtual Integration. IEEE Des. Test.

[24] Plourde J., Arney D., Goldman J.M. OpenICE: An open, interoperable platform for medical cyber-physical systems. Proceedings of the 2014 ACM/IEEE International Conference on Cyber-Physical Systems (ICCPS).

[25] Ivanov R., Nguyen H., Weimer J., Sokolsky O., Lee I. OpenICE-lite: Towards a Connectivity Platform for the Internet of Medical Things. Proceedings of the 2018 IEEE 21st International Symposium on Real-Time Distributed Computing (ISORC).

[26] Mahadevan P., Krioukov D., Fomenkov M., Huffaker B., Dimitropoulos X., Claffy K., Vahdat A. (2006). The Internet AS-Level Topology: Three Data Sources and One Definitive Metric. ACM SIGCOMM Comput. Commun. Rev..

[27] Tramarin F., Narduzzi C., Vitturi S., Bertocco M. (2018). A Calibrated Test-Set for Measurement of Access-Point Time Specifications in Hybrid Wired/Wireless Industrial Communication. Information.

[28] De Vito L., Rapuano S., Tomaciello L. (2008). One-Way Delay Measurement: State of the Art. IEEE Trans. Instrum. Meas..

[29] Ferrari P., Sisinni E., Brandao D., Rocha M. Evaluation of communication latency in industrial IoT applications. Proceedings of the 2017 IEEE International Workshop on Measurements and Networking (M&N).

[30] Ferrari P., Flammini A., Rinaldi S., Sisinni E. Evaluation of Communication Delay in IoT Applications Based on OPC UA. Proceedings of the 2018 Workshop on Metrology for Industry 4.0 and IoT.

[31] Fernandes Carvalho D., Ferrari P., Flammini A., Sisinni E. A Test Bench for Evaluating Communication Delays in LoRaWAN Applications. Proceedings of the 2018 Workshop on Metrology for Industry 4.0 and IoT.

[32] Silva D., Oliveira G., Silva I., Ferrari P., Sisinni E. Latency Evaluation for MQTT and WebSocket Protocols: An Industry 4.0 Perspective. Proceedings of the 2018 IEEE Symposium on Computers and Communications (ISCC).

[33] Ferrari P., Flammini A., Rinaldi S., Sisinni E., Maffei D., Malara M. (2018). Impact of Quality of Service on Cloud Based Industrial IoT Applications with OPC UA. Electronics.

[34] Ferrari P., Flammini A., Sisinni E., Rinaldi S., Brandao D., Rocha M. (2018). Delay Estimation of Industrial IoT Applications Based on Messaging Protocols. IEEE Trans. Instrum. Meas..

[35] Tobagi F.A., Markopoulou A.P., Karam M.J. Is the Internet ready for VoIP?. Proceedings of the IWDC 2002.

[36] Collina M., Bartolucci M., Vanelli-Coralli A., Corazza G.E. Internet of Things application layer protocol analysis over error and delay prone links. Proceedings of the 7th Advanced Satellite Multimedia Systems Conference and the 13th Signal Processing for Space Communications Workshop (ASMS/SPSC).

[37] Govindan K., Azad A.P. End-to-end service assurance in IoT MQTT-SN. Proceedings of the 12th Annual IEEE Consumer Communications and Networking Conference (CCNC).

[38] Mijovic S., Shehu E., Buratti C. Comparing application layer protocols for the Internet of Things via experimentation. Proceedings of the IEEE 2nd International Forum on Research and Technologies for Society and Industry Leveraging a better tomorrow (RTSI).

[39] Pereira C., Pinto A., Ferreira D., Aguiar A. (2017). Experimental Characterisation of Mobile IoT Application Latency. IEEE Internet Things J..

[40] Rinaldi S., Pasetti M., Sisinni E., Bonafini F., Ferrari P., Rizzi M., Flammini A. (2018). On the Mobile Communication Requirements for the Demand-Side Management of Electric Vehicles. Energies.

[41] Sherman J.A., Levine J. (2016). Usage Analysis of the NIST Internet Time Service. J. Res. Natl. Inst. Stand. Technol. Gaithersburg.

[42] Angrisani L., Capriglione D., Ferrigno L., Miele G. (2013). Internet Protocol Packet Delay Variation measurements in communication networks: How to evaluate measurement uncertainty?. Meas. J. Int. Meas. Conf..

